# Methylation profiling of *SOCS1*, *SOCS2*, *SOCS3*, *CISH* and *SHP1* in Philadelphia-negative myeloproliferative neoplasm

**DOI:** 10.1111/jcmm.12103

**Published:** 2013-10-16

**Authors:** Min Yue Zhang, Tsz Kin Fung, Fang Yuan Chen, Chor Sang Chim

**Affiliations:** a Department of Hematology, Ren Ji Hospital, Shanghai Jiaotong University School of MedicineShanghai, China; b Department of Medicine, Queen Mary Hospital, The University of Hong KongPokfulam, Hong Kong

**Keywords:** myeloproliferative neoplasms, methylation, *SOCS1*, *SOCS2*, *SOCS3*, *CISH*, *SHP1*, MSP primer

## Abstract

Janus kinase-signal transducer and activator of transcription (JAK/STAT) signalling, pivotal in Philadelphia-negative (Ph-ve) myeloproliferative neoplasm (MPN), is negatively regulated by molecules including SOCSs, CISH and SHP1. *SOCS1*, *SOCS2* and *SOCS3* methylation have been studied in MPN with discordant results. Herein, we studied the methylation status of *SOCS1*, *SOCS2* and *SOCS3*, *CISH* and *SHP1* by methylation-specific polymerase chain reaction (MSP) in cell lines and 45 diagnostic marrow samples of Ph-ve MPN. Moreover, we attempted to explain the discordance of methylation frequency by mapping the studied MSP primers to the respective genes. Methylation was detected in normal controls using *SOCS2* MSP primers in the 3′translated exonic sequence, but not primers around the transcription start site in the 5′ untranslated regions (5′UTR). *SOCS1*, *SOCS2*, *SOCS3* and *CISH* were completely unmethylated in primary MPN samples and cell lines. In contrast, methylation of *SHP1* was detected in 8.9% primary marrow samples. Moreover, *SHP1* was completely methylated in K562 cell line, leading to reversible *SHP1* silencing. A review of methylation studies of *SOCS1* and *SOCS3* showed that spuriously high rates of *SOCS* methylation had been reported using MSP primers targeting CpG sites in the 3′translated exonic sequence, which is also methylated in normal controls. However, using MSP primers localized to the 5′UTR, methylation of *SOCS1, SOCS2* and *SOCS3* is infrequent across all studies. In summary, methylation of *SOCS1*, *SOCS2*, *SOCS3* and *CISH* is infrequent in Ph-ve MPN. Appropriate MSP primers are important for accurate estimation of the methylation frequency. The role of *SHP1* methylation in the pathogenesis of MPN warrants further investigation.

## Introduction

Philadelphia-negative (Ph-ve) myeloproliferative neoplasm (MPN) is a stem cell disease with proliferation of myeloid compartment, leading to development of distinct clinical entities such as polycythemia vera (PV), essential thrombocythemia (ET) and primary myelofibrosis (PMF) [1, 2]. Recently, *Janus kinase 2 (JAK2) V617F* mutation was detected in most of the patients with PV and about half of the patients with ET and PMF, leading to constitutive activation of Janus kinase-signal transducer and activator of transcription (JAK/STAT) signalling [3, 4]. However, pathogenesis in those without *JAK2 V617F* mutation remains unknown.

The JAK/STAT signalling pathway is important for the transmission of cytokine signals from cell surface to the nucleus [5]. Binding of cytokines to their cognate receptors results in the dimerization of receptor complexes and activation of the Janus family of protein tyrosine kinases [6, 7], followed by phosphorylation of the cytoplasmic STATs. Upon phosphorylation, STATs form homo- or hetero-dimers migrate to the nucleus and activate gene transcription. This JAK/STAT pathway is subject to negative regulation by PIAS members, JAK inhibitors like SOCS family proteins and protein tyrosine phosphatases (PTP) such as SHP1 [6–8]. The *SOCS* family comprises eight members, including *SOCS1-7* and *CISH*, and is characterized by the presence of a central Src homology (SH2) domain that is flanked by a variable length N-terminal domain, and a conserved 40 amino acid carboxy terminal ‘SOCS box’ domain [6, 7]. SOCS proteins suppress JAK/STAT signalling by binding with their SH2 domain to phosphotyrosine residues in cytokine receptors or activated JAKs [9]. Among them, *CISH*, localized at 3p21.2; *SOCS1*, at 16p13.13; *SOCS2* at 12q22; and *SOCS3* at 17q25.3 are most thoroughly studied in haematological malignancies. SOCS1 and SOCS3 can bind the activated JAKs and cytokine receptors, respectively, through SH2 domain to inhibit JAK tyrosine kinase activation. CISH and SOCS2 inhibit STAT activation by binding to phosphorylated tyrosine residues on activated cytokine receptors and compete with STAT or hinder the STAT-binding sites of receptors [9]. SOCS members are cytokine-inducible negative regulators of the cytokine signalling [9]. *SOCS1, SOCS2, SOCS*3 and *CISH* can be induced by a multitude of cytokines, and hence serve as negative feedback to curb excessive cytokine signalling [9]. *SOCS1* and *SOCS3* comprise two exons with the first exon being untranslated, whereas *SOCS2* consists of three exons with the first exon being untranslated [10–12]. All these three genes are embedded in a huge CpG island of more than two kilobases extending from the 5′UTR to the 3′translated exonic sequence, as demonstrated in the corresponding websites in NCBI NIH Roadmap Epigenomics project [13].

SHP1, also known as HCP, SHPTP1 and PTP1C, is a 68-kd, cytoplasmic PTP [14]. The human *SHP1* gene is located on chromosome 12p13, consists of 17 exons and spans ∼17 KB of DNA. It contains two tandem Src homology (SH2) domains, a catalytic domain and a C-terminal tail of about 100 amino acid residues [14]. In contrast to the ubiquitous expression of the structurally related *SHP2*, *SHP1* is primarily expressed in haematopoietic cells, and considered a putative tumour suppressor gene in lymphoma and leukaemia, as it antagonizes the growth-promoting and oncogenic potentials of protein tyrosine kinase [14].

Hypermethylation of promoter-associated CpG islands of tumour suppressor genes and recently microRNA [15–17], resulting in gene silencing, and hence inactivation of tumour suppressor genes, has been implicated in the pathogenesis of haematological malignancies [18]. Moreover, aberrant DNA methylation of *SOCS1*, *SOCS2*, *SOCS3* and *SHP1* has been studied in Ph-ve MPN with discordant results [19–23]. Our previous study has shown that while normal cells possess unmethylated CpG islands around the transcription start site (TSS) in the 5′UTR, CpG sites inside the coding exonic sequence are methylated for *SOCS1*, thereby emphasizing the importance of methylation-specific polymerase chain reaction (MSP) primer selection [24, 25]. Moreover, there is scanty data on the methylation of *CISH* in Ph-ve MPN. In this study, we investigated the methylation profile of *SOCS1*, *SOCS2*, *SOCS3*, *CISH* and *SHP1* in MPN by the use of MSP primers in the both 5′UTR and translated exonic sequence of the genes. Finally, we attempted to explain the discordance of methylation frequency of *SOCS1* and *SOCS3* in MPN by mapping the studied MSP primers to the respective genes.

## Materials and methods

### Patient samples

DNA was extracted from primary bone marrow samples at diagnosis of 45 patients with Ph-ve MPN. Diagnosis of Ph-ve MPN including ET, PV and PMF was based on WHO criteria. MSP on DNA extracted from primary marrow samples at diagnosis was performed on 45 patients with MPN [ET, *N* = 34 (75.6%); PV, *N* = 7 (15.6%) and PMF, *N* = 4 (8.9%)]. The clinical features of these patients have been previously described [26–28]. In brief, there were 24 (53.5%) male and 21 (46.7%) female patients with a median age of 67.5 years (range: 28–89 years) The median presenting platelet count of 848 × 10^9^/l, presenting Hb of 9–22 g/dl (median 13.3 g/dl), median presenting leucocyte count of 14.4 × 10^9^/l (range: 7–28 × 10^9^/l). Apart from 5 (11.1%) patients in whom the presenting symptoms was unavailable, 25 (62.5%) were asymptomatic at diagnosis, 4 (10%) with bleeding, 4 (10%) with erythromelalgia, 2 (5%) with minor stroke, 3 (7.5%) with abdominal pain and one each (2.5%) with blurred vision and weight loss. JAK2 V617F mutation was presented in 26 of the 40 patients. DNA from three normal bone marrow donors and five normal peripheral blood donors was used as negative control, while enzymatically methylated control DNA (CpGenome Universal Methylated DNA, Millipore, Billerica, MA, USA) was considered as positive control. The study has been approved by Institutional Review Board of Queen Mary Hospital, and written informed consent has been obtained.

### Cell lines and culture

Cell lines and culture were described in previous study [28]. SET-2 cells were purchased from Deutsche Sammlung von Mikroorganismen und Zellkulturen GmbH (DMSZ) (Braunschweig, Germany). HEL cells is a human erythroleukemia cell line generously provided by Dr Zhang Dong-Er, Department of Pathology and Molecular Biology, Moores Cancer Center, University of California San Diego, USA. MEG-01 and K562 cells were kindly provided by Dr Mo Yang, Department of Paediatrics, Queen Mary Hospital, The University of Hong Kong, Hong Kong. SET-2 was derived from ET at megakaryoblastic leukaemic transformation. HEL was derived from peripheral blood of a patient with erythroleukemia. Both SET-2 and HEL carried JAK2 V617F mutation. MEG-01 and K562 were derived from blastic transformation of patients with CML and carried *BCR/ABL* fusion gene. Cell cultures were cultured in RPMI-1640 medium (Invitrogen, Carlsbad, CA, USA), supplemented with 10% foetal bovine serum (20% for SET-2; Invitrogen), 50 U/ml penicillin and 50 μg/ml streptomycin (Invitrogen) in a humidified atmosphere of 5% CO_2_ at 37°C.

### Methylation-specific polymerase chain reaction

DNA extraction from bone marrow samples, peripheral blood and cell lines was performed with QIAamp DNA Blood Mini Kit according to the manufacturer's instructions. DNA bisulphite conversion was performed with a commercially available kit (EpiTect Bisulfite Kit, Qiagen, Duesseldorf, Germany). Methylation status of *SOCS1, SOCS2, SOCS3*, *CISH* and *SHP1* gene promoter CpG islands was investigated by MSP as previously reported [24]. Primers used for the methylated MSP (M-MSP) and unmethylated MSP (U-MSP) were listed in the Table S1. For *SOCS1*, based on our previous study [24], MSP primer inside exon 1 was methylated in normal controls, and hence unsuitable for methylation analysis. In contrast, MSP primers upstream of the translation start region (MSP-5′) were unmethylated in normal controls, and hence used in this study. For *SOCS2*, two sets of primers, located 5′ (*SOCS2*-5′) and 3′ (*SOCS2-*3′) to the translation start site of *SOCS2* were used in this study. *SOCS2-*5′ primers were used according to previous study [29]. *SOCS2-*3′ primers were mapped to the region, in which methylation of *SOCS2* has been studied by bisulphite genomic sequencing and methylation-sensitive restriction enzyme assay in MPN patients samples by Quentmeier *et al*. [30]. *SOCS3* primers were adopted from He *et al*. [31]. All MSP were performed in thermal cycler (9700, Applied Biosystems, Foster City, CA, USA) under the conditions: 95°C for 10 min., followed by specific cycles of 95°C for 30 sec., specific annealing temperature for 30 sec., 72°C for 30 sec. and a final extension of 10 min. at 72°C (Table S1). The MSP mixture contained 30 ng of bisulphite-treated DNA, 0.2 mM dNTPs, MgCl_2_ (Table S1), 10 pmol of each primer, 1× PCR buffer and 1 unit of FastStart Taq DNA polymerase (Roche, Mannheim, Germany) in a final volume of 25 μl. Ten microlitre of PCR products was loaded onto 6% polyacrylamide gels, electrophoresed, stained with ethidium bromide and visualized under ultraviolet light.

### 5-Aza-2′-deoxycytidine (5-AzadC) treatment of the cell line K562

K562 cells were completely methylated for *SHP1*. For treatment with 5-AzadC, K562 cells (1 × 10^6^ cells/ml) were seeded into six-well plates on day 0 and cultured with 0.5 μM of 5-AzadC from day 1 to day 3. Cells on day 0 and day 3 of treatment were harvested.

### Reverse transcription-PCR for SHP1

MirVana™ miRNA Isolation Kit (Ambion, Austin, TX, USA) and QuantiTect Reverse Transcription kit (Qiagen, Duesseldorf, Germany) were used for isolation of total RNA and RT-PCR, respectively, according to the manufacturers’ instructions. For *SHP1* RT–PCR, the following primers: forward 5′-GGC ACT GGG AGC TGC ATC TGA GGC-3′; reverse 5′-CTC GCA CAT GAC CTT GAT GTG-3′ were used. For *GAPDH* RT–PCR, the following primers: forward 5′-ACC ACA GTC CAT GCC ATC ACT-3′; reverse 5′-TCC ACC ACC CTG TTG CTG TA-3′ were used.

### Statistical analysis

Correlation between *SHP1* gene methylation status and the presence of *JAK2 V617F* mutation was computed by the Chi-square test (or Fisher Exact test) using Statistical Package for the Social Sciences (SPSS, IBM, New York, NY, USA) version 18.0. All *P* values were two-sided.

## Results

### Controls

**Fig. 1:**
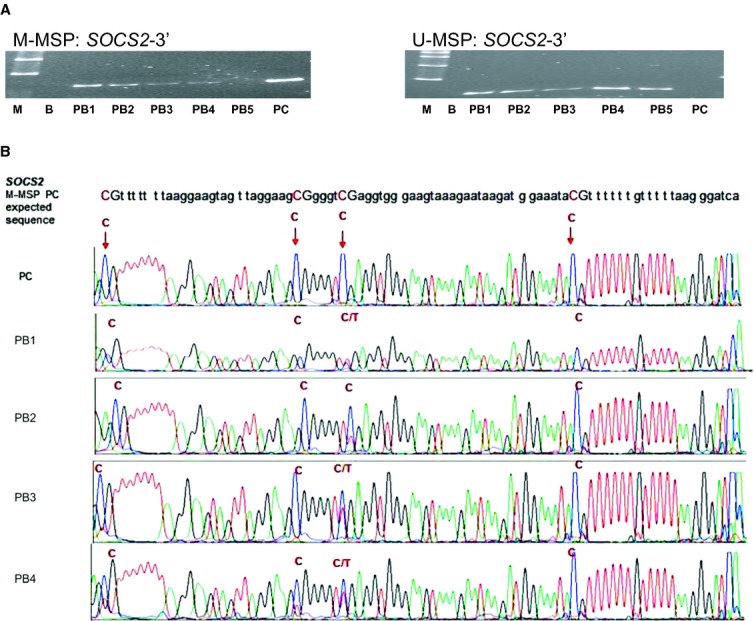
(**A**) Methylation-specific PCR of normal controls using *SOCS2*-3′ methylation-specific polymerase chain reaction (MSP) primers. M-/U-MSP analysis showed that four of five normal peripheral blood controls (PB1-5) were methylated with the *SOCS2*-3′ primers. (M: DNA marker; B: blank; PC: positive control; PB, normal peripheral blood control). (**B**) Sequencing of MSP products using *SOCS2*-3′ MSP primers in normal controls and positive control showing methylation signals. Methylated cytosine residues [C] in CpG dinucleotide remained as C, whereas unmethylated cytosine read as [T] after bisulphite conversion. (PC: positive control; PB: normal peripheral blood control).

**Fig. 2:**
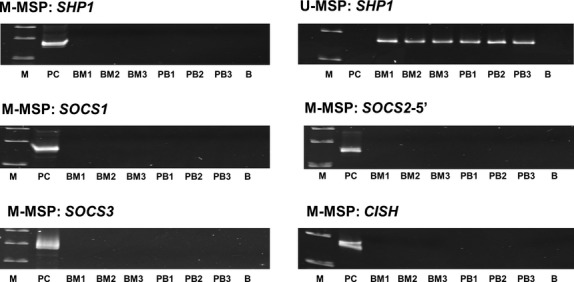
Methylation-specific polymerase chain reaction (MSP) of *SOCS1*, *SOCS2*-5′, *SOCS3*, *CISH* and *SHP1* in normal controls. M-/U-MSP analysis showed that three normal peripheral blood controls (PB1-3) and three bone marrow controls (BM1-3) were unmethylated with the *SOCS1*, *SOCS2*-5′, *SOCS3*, *CISH* and *SHP1* primers. (M: DNA marker; B: blank; PC: positive control; PB, normal peripheral blood control; BM: normal bone marrow control).

As mentioned in the section for materials and methods, two sets of MSP primers, one in the 5′UTR and the other in the 3′translated exon sequence, were available for *SOCS1* (*SOCS1*-5′ and *SOCS1*-3′) and *SOCS2* (*SOCS2*-5′ and *SOCS2*-3′). *SOCS1*-3′ has been shown to be methylated in normal controls [24], and hence *SOCS1*-5′ MSP primers was used in this study. Using the MSP primers in the 5′UTR near the TSS as described previously, *SOCS1* was unmethylated in normal controls, but methylated in the positive control [24]. For *SOCS2*, using downstream MSP primers inside the translated exonic sequence, i.e. *SOCS2*-3′, methylation was detected in four of five normal control DNA samples (Fig. 1A). Moreover, sequencing of the M-MSP products confirmed authenticity of methylation in these normal controls, which was demonstrated by the presence of unconverted, and hence methylated, cytosine molecules at ‘CpG’ dinucleotide (Fig. 1B). On the other hand, using MSP primers in the 5′UTR near the TSS, *SOCS2*-5′ MSP primers, *SOCS2* was unmethylated in all of the normal control samples, but methylated in the positive control DNA (Fig. 2). Therefore, we studied methylation of the *SOCS2* gene using MSP with primer set in the 5′UTR near the TSS instead of inside the translated exon sequence. For the *SOCS3*, *SHP1* and *CISH* genes, M-MSP was negative in normal DNA, while positive for the methylated control DNA. Conversely, U-MSP was positive in normal DNA, while negative for the methylated control DNA (Fig. 2). Sequencing of the M-MSP products of these four genes from the methylated control DNA showed the expected nucleotide changes, and hence confirmed complete bisulphite conversion and specificity of MSP primers.

### Cell lines

**Fig. 3:**
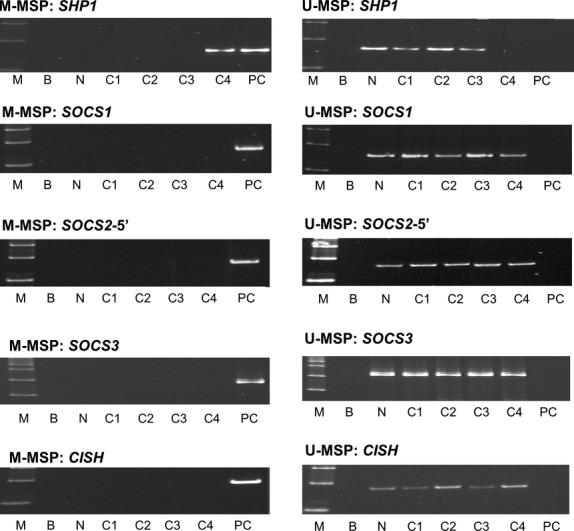
Methylation-specific polymerase chain reaction of *SOCS1*, *SOCS2*-5′, *SOCS3, CISH* and *SHP1* in cell lines. For *SHP1*, K562 was homozygously methylated, while HEL, SET-2 and MEG-01 were completely unmethylated. HEL, SET-2, MEG-01 and K562 were completely unmethylated for *SOCS1*, *SOCS2*-5′, *SOCS3* and *CISH*. (M: DNA marker; B: blank; PC: positive control; N: normal control; C1: HEL; C2: MEG-01; C3: SET-2; C4: K562).

*SHP1* was homozygously methylated in K562, but completely unmethylated in HEL, MEG-01 and SET-2. On the other hand, *SOCS1*, *SOCS2*, *SOCS3* and *CISH* genes were completely unmethylated in these four cell lines (Fig. 3).

### Primary samples

**Fig. 4:**
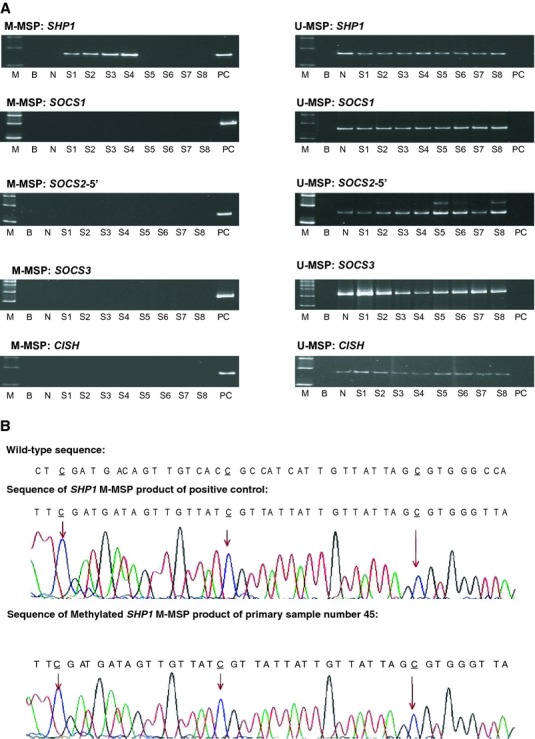
Methylation-specific polymerase chain reaction (MSP) of *SOCS1*, *SOCS2*-5′, *SOCS3*, *CISH* and *SHP1* in MPN primary samples. (**A**) M-/U-MSP analysis showed that for *SOCS1*, *SOCS2*-5′, *SOCS3*, *CISH*, methylation was absent in MPN marrow samples, whereas for *SHP1*, methylation was found in four MPN patients. (M: DNA marker; B: blank; PC: positive control; N: normal control; S: primary sample). (**B**) DNA sequencing of *SHP1* M-MSP products from bisulphite-converted methylated positive and MPN primary samples showing methylated cytosine [C] residues in CpG dinucleotide remained unchanged, unmethylated C residues were converted into [T], whereas all the non-CpG C residues were unmethylated and were converted to thymidine [T].

Methylation-specific polymerase chain reaction was performed in 45 primary samples of Ph-ve MPN patients for these five genes. None of the patients carried hypermethylation of *SOCS1*, *SOCS2*, *SOCS3* and *CISH* (Fig. 4A). Nevertheless, methylation of *SHP1* was found in four ET patients, which was confirmed by direct sequencing of M-MSP products of positive control and primary samples (Figs 4A and B). Of these, three (75%) patients carried *JAK2 V617F* mutation. Two were female. The median age was 63.3 years. There was no association of *SHP1* methylation and JAK2 V617F mutation (*P* = 0.99). One presented with cerebral infarction, one with peptic ulcer and the other two were asymptomatic at diagnosis. The median diagnostic Hb was 12.7 g/dl, platelet 962 × 10^9^/L and leucocyte 15.4 × 10^9^/L. Median survival was 48.6 months. At the time of writing, two had died, one of cerebral infarction and the cause of death of the other was unknown. No myeloid transformation had occurred.

### 5-AzadC treatment of K562 cells

**Fig. 5:**
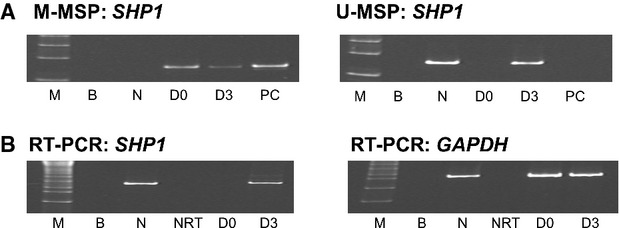
Effect of 5-AzadC treatment on K562 cells. (A) M-/U-methylation-specific polymerase chain reaction analysis of *SHP1* promoter methylation status showed that 5-AzadC treatment led to progressive demethylation of *SHP1* promoter in K562 cells. (M: DNA marker; B: blank; PC: positive control; N: normal control; D0, day 0; D3, day 3 culture in 5-AzadC with 0.5 μM). (B) Reverse transcription–PCR (RT-PCR) analysis of the *GAPDH* status and *SHP1* expression after 5-AzadC treatment. (M: DNA marker; B: blank; N: normal control; NRT: negative control without reverse transcriptase; D0, day 0; D3, day 3 culture in 5-AzadC with 0.5 μM).

Untreated K562 cell line showed complete methylation of *SHP1*. Treatment of cells with 0.5 μM 5-AzadC for 3 days led to demethylation of *SHP1* as demonstrated by the emergence of *SHP1* U-MSP signal (Fig. 5A). Moreover, RT–PCR demonstrated that *SHP1* gene was re-expressed after 5-AzadC treatment of K562 cells (Fig. 5B).

## Discussion

To account for the discrepancy among the frequency of methylation of *SOCS1*, *SOCS2* and *SOCS3* in MPN, we have mapped the MSP primers used in different studies onto the gene map of *SOCS1*, *SOCS2* and *SOCS3* (Figures S1–3; Table 1) and derived the following observations.

**Table 1:** Literature about the frequencies of *SOCS1, SOCS2, SOCS3*, *SHP1* and *CISH* methylation in Ph-ve MPN. Primers positions of *SOCS1*, *SOCS2* and *SOCS3* genes are represented by arrows in supplementary figures

Author	Zhang	Jost [23]	Fourouclas [22]	Capello [20]	Quentmeier* [30]	Fernandez -Mercado [21]	Teofili [29]	Barrio† [19]	Fodermayr [34]
No of case	46	39	73	112	7	39	81	35	39
Genes studied	*SOCS1, SOCS2, SOCS3*, *CISH*, *SHP1*	*SOCS1*, *SHP1*	*SOCS1*, *SOCS3*	*SOCS1*, *SOCS3*, *SHP1*	*SOCS2*	*SOCS1*, *SOCS3*	*SOCS1, SOCS2, SOCS3*,	*SOCS1, SOCS2, SOCS3*,, *CISH*	*SOCS1*, *SOCS3*, *SHP1*
*SOCS1* Primer site	5′ UTR: Figure S1-A	exon2: Figure S1-C	5′ UTR: Figure S1-B exon2: Figure S1-C	exon2: Figure S1-C	—	5′ UTR: Figure S1-A exon2 : no mention	exon2: Figure S1-C	No mention	exon2: Figure S1-C
% *SOCS1* methylation	0	15.4	5′ UTR : 0; exon2: 38.4	13.4	—	5′ UTR : 0 exon2 region 1: 72.2 exon2 region 2: 0	13.5	34.3	12.8
*SOCS2* Primer site	5′ UTR: Figure S2-A	—	—	—	5′ UTR: Figure S2-B	—	5′ UTR: Figure S2-A	No mention	—
% *SOCS2* methylation	0	—	—	—	28.6	—	0	0	—
*SOCS3* Primer site	5′ UTR: Figure S3-C	—	5′ UTR: Figure S3-A	exon 2:Figure S3-D	—	5′UTR: Figure S3-B intron 1	5′ UTR: Figure S3-C	No mention	Intron 1
% *SOCS3* methylation	0	—	None of PV or ET but 27% of PMF	41.1	—	5′UTR: 7.7 intron 1: 23.1	13.5	0	15.4
% SHP-1 methylation	8.7	0	—	7.1	—	—	—	—	12.8
% *CISH* methylation	0	—	—	—	—	—	—	0	—

* Using bisulphite genomic sequencing and methylation-sensitive restriction enzyme assay to detect methylation status.

† Using Human-Methylation27 DNA Analysis BeadChip’ (Illumina) to detect methylation status.

UTR: untranslated region; PV: polycythemia vera; ET: essential thrombocytosis; PMF: primary myelofibrosis.

Firstly, *SOCS1*, *SOCS2* and *SOCS3* genes have multiple exons with the first exon being untranslated. Moreover, all of them are embedded in a huge CpG island spanning >2 kb that extends from the 5′UTR into the 3′translated protein-coding exonic sequences (Figures S1–3).

In a previous study, we have shown that using MSP primers of *SOCS1-3′* located in the translated exon 2, as described by Watanabe *et al*. [32], methylation was detected in six of 12 normal peripheral blood and two of three normal marrow samples [24]. These results were confirmed by sequencing, suggesting that methylation within *SOCS1* exon 2 might not be involved in the regulation of gene transcription. The lack of impact of *SOCS1* exon 2 methylation on gene expression has been illustrated in HL60 and U937 cell lines, in which complete methylation of CpG islands within *SOCS1* exon 2 was associated with significant expression of *SOCS1* [32] (Figure S1), and hence a lack of correlation between methylation of CpG sites in the 3′translated exonic sequence and gene silencing. On the contrary, methylation of CpG islands in the 5′UTR, using MSP primer targeting CpG sites in the 5′UTR, has been shown to be associated with inhibition of expression of, and hence silencing of *SOCS1* by immunohistochemistry in hepatoblastoma [33]. Therefore, methylation of *SOCS1* should be studied with MSP primers mapping to the 5′UTR (*SOCS1*-5′) as performed in this study. Using this *SOCS1*-5′ MSP primers, *SOCS1* methylation was not detected in any primary MPN samples or cell lines. Similarly, other studies using primers targeting CpG sites in the 5′UTR also showed that none of a total of 112 MPN cases (73 in Fourouclas *et al*. and 39 in Fernández-Mercado *et al*.) had methylation of *SOCS1* [21, 22] (Figure S1). In contrast, studies using MSP primers inside the 3′translated exonic sequence showed methylation frequencies ranging from 12.8% to 72% (median: 14.5%) [20–23, 29, 34] (Figure S1). Therefore, these findings were consistent with the notion that a boundary exists between methylated and unmethylated CpG dinucleotide within a CpG island, and that CpG dinucleotide inside the 3′translated protein-coding sequences is commonly methylated in normal cells.

Similarly, *SOCS2* methylation has been reported to be frequently methylated in MPN [30]. In contrast, using MSP primers near the TSS in the 5′UTR, we and others found the absence of *SOCS2* methylation [19, 29]. Quentmeier *et al*., using methylation-sensitive enzymes targeting CpG sites inside the 3′translated exon 2 sequence, showed that 28.6% of MPN patients had methylation of *SOCS2* [30] (Figure S2). In this study, we have designed MSP primers mapping to the same exon 2 sequence, and confirmed that CpG sites in this region is in fact methylated in normal controls, and hence unimportant for epigenetic regulation of *SOCS2* transcription, and unsuitable for methylation study.

Moreover, using MSP primers close to the TSS in the 5′UTR (Figure S3), we and others showed infrequent methylation of *SOCS3* in MPN (this study, 0%; Fourouclas *et al*., 27%; Teofilli *et al*., 13.5% and Fernández-Mercado *et al*., 7.7%) [21, 22, 29], in contrast to frequent methylation of 41.1% samples when MSP primers inside the translated exonic DNA sequence were used [20]. In our study, we adopted MSP primers from He *et al*. [31] near the TSS (Figure S3) for several reasons. Firstly, our MSP primers were located close to the TSS in the 5′UTR region. Secondly, methylation in CpG islands within this region has been shown to be associated with *SOCS3* silencing and aberrant activation of JAK/STAT signalling in lung cancer cell lines, and hence biologically relevant [31]. Using MSP primers in the 5′UTR, *SOCS3* methylation was not detected in any of the patients with PV or ET [22]. However, Fourouclas *et al*. demonstrated *SOCS3* methylation in 27% patients with PMF, suggesting a disease-specific *SOCS3* methylation among MPN [22]. Unfortunately, in our series, we had too few patients with PMF to verify this finding.

Overall, our findings and literature review showed that MSP primer selection is important in the study of methylation as a methylation boundary may occur in some genes, in which methylation of CpG inside the translated exonic DNA sequence, unrelated to gene silencing, may occur in normal cells. Indeed, in a study of the *HIC1* gene, CpG island methylation inside exon 3 was present in normal controls, which was not associated with down-regulation of *HIC1*, and hence unimportant for transcriptional regulation of *HIC1*. In contrast, CpG sites in the 5′UTR was unmethylated in normal marrow control, but aberrantly methylated in some AML samples, thereby emphasizing the importance of methylation of 5′UTR, promoter-associated CpG islands, instead of downstream CpG sites in the translated exonic region in epigenetic regulation of gene expression [35]. Moreover, a possible boundary between methylated and unmethylated sequence in *SOCS1* gene was suggested by the observation that methylation was absent in the 5′UTR of *SOCS1*, but was detected in 85% of normal DNA using MSP primers targeting CpG sites in the 3′translated exon 2 sequence [21]. Similarly, in *SOCS3*, methylation was infrequent using primers near the TSS in the 5′UTR, but present in 41% of primary MPN samples when MSP primers inside the translated exon 2 sequence were used [20–22, 29]. Therefore, one has to select MSP primers in the 5′UTR region, particularly close to the TSS, or else, methylation unrelated to epigenetic regulation might give rise to spuriously high frequency of gene methylation.

In contrast to the *SOCS* family, *SHP1*, another negative regulator of JAK/STAT signalling, has been infrequently studied for methylation in MPN. We have shown that in myeloma, *SHP1* methylation was frequent, leading to reversible *SHP1* silencing and constitutive JSK/STAT activation [24]. Here, we showed methylation of *SHP1* in primary MPN samples and K562 cells, which were verified by direct sequencing of M-MSP PCR products. Therefore, *SHP1* methylation, leading to reversible gene silencing, is implicated in the constitutive activation of JAK/STAT in MPN, and hence warrants further study in larger number of patients. Similarly, *CISH* methylation is absent in MPN.

## Conclusion

In conclusion, this is a comprehensive study of methylation profile of *SOCS1*, *SOCS2*, *SOCS3*, *CISH* and *SHP1* in MPN. Among these, only *SHP1* was methylated in MPN, and hence implicated in the constitutive activation of JAK/STAT signalling in MPN. Moreover, careful selection of MSP primers is important.

## Supporting information

**Figure S1** Schematic diagram of the *SOCS1* gene and the positions of primers for MSP. Primers positions are represented by arrows.

**Figure S2** Schematic diagram of the *SOCS2* gene and the positions of primers for MSP. Primers positions are represented by arrows.

**Figure S3** Schematic diagram of the *SOCS3* gene and the positions of primers for MSP. Primers positions are represented by arrows.

**Table S1** MSP primer sequences and reaction conditions.
