# Supplementary figures and images for: Methylation profiling of SOCS1, SOCS2, SOCS3, CISH and SHP1 in Philadelphia-negative myeloproliferative neoplasm
Source: J Cell Mol Med. 2013 Oct 16;17(10):1282–90. doi: 10.1111/jcmm.12103 (PMC4159021; doi:10.1111/jcmm.12103)

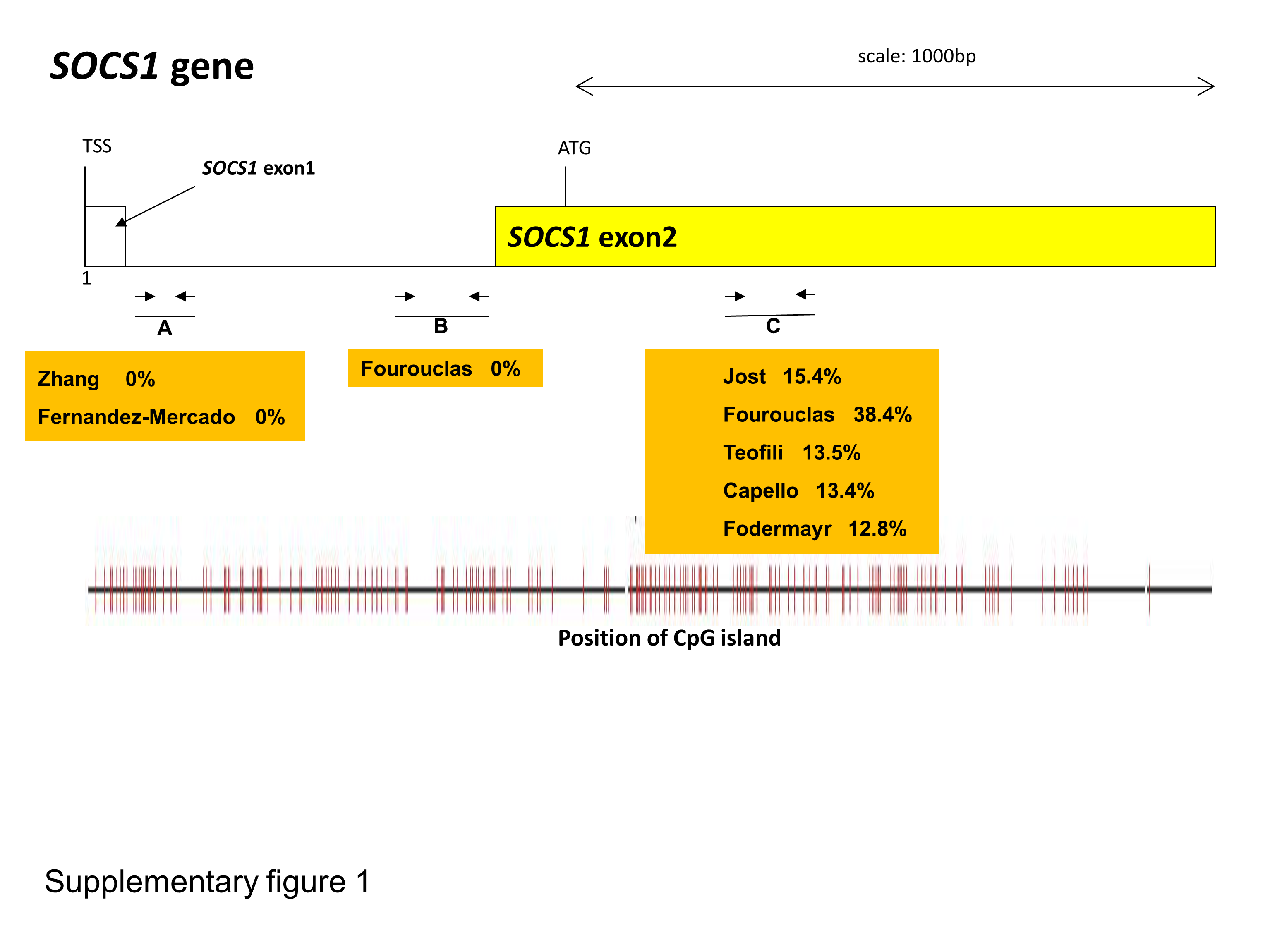

Supplement: Supplementary file 1 [file jcmm0017-1282-SD1.tif]

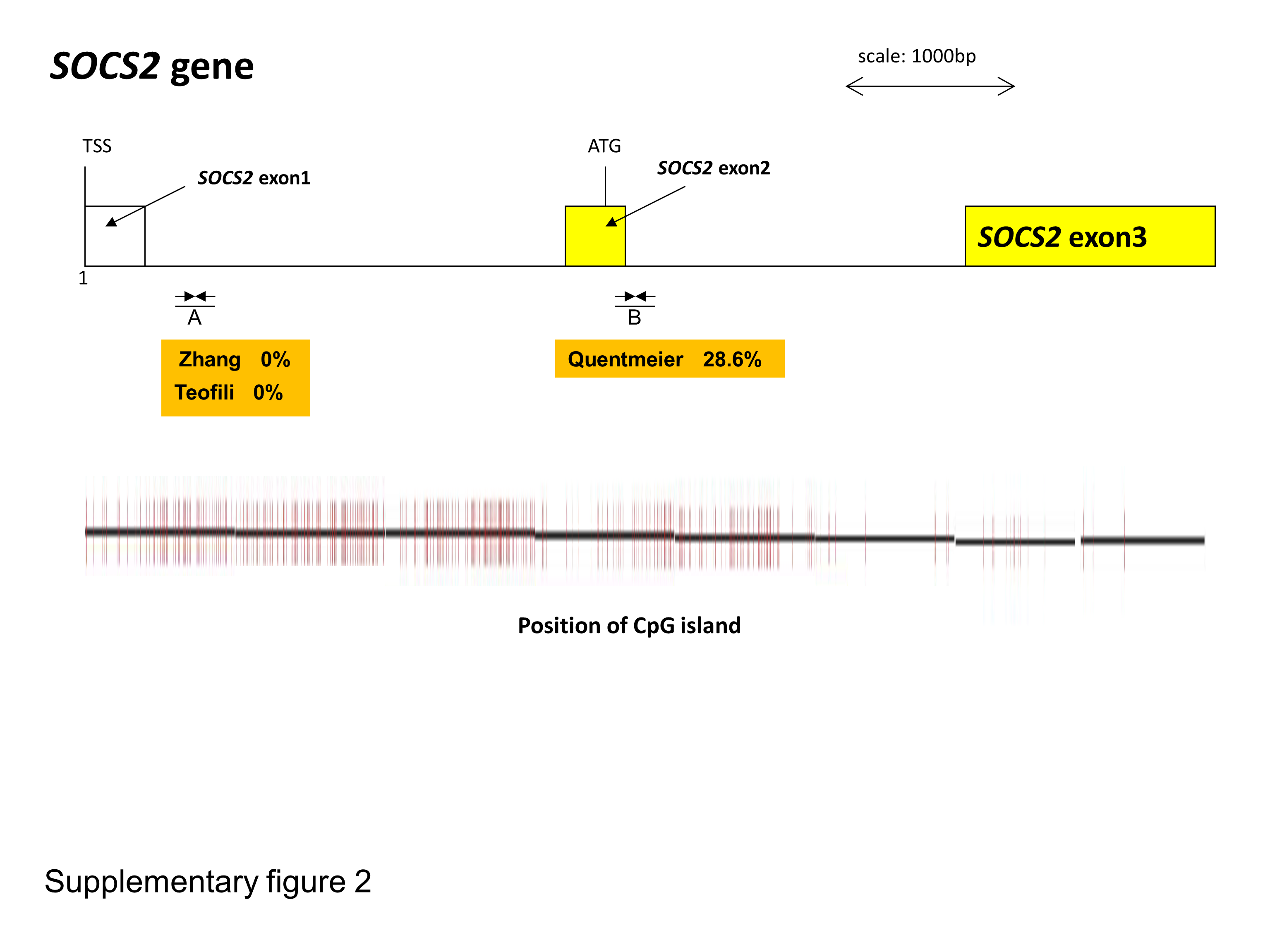

Supplement: Supplementary file 2 [file jcmm0017-1282-SD2.tif]

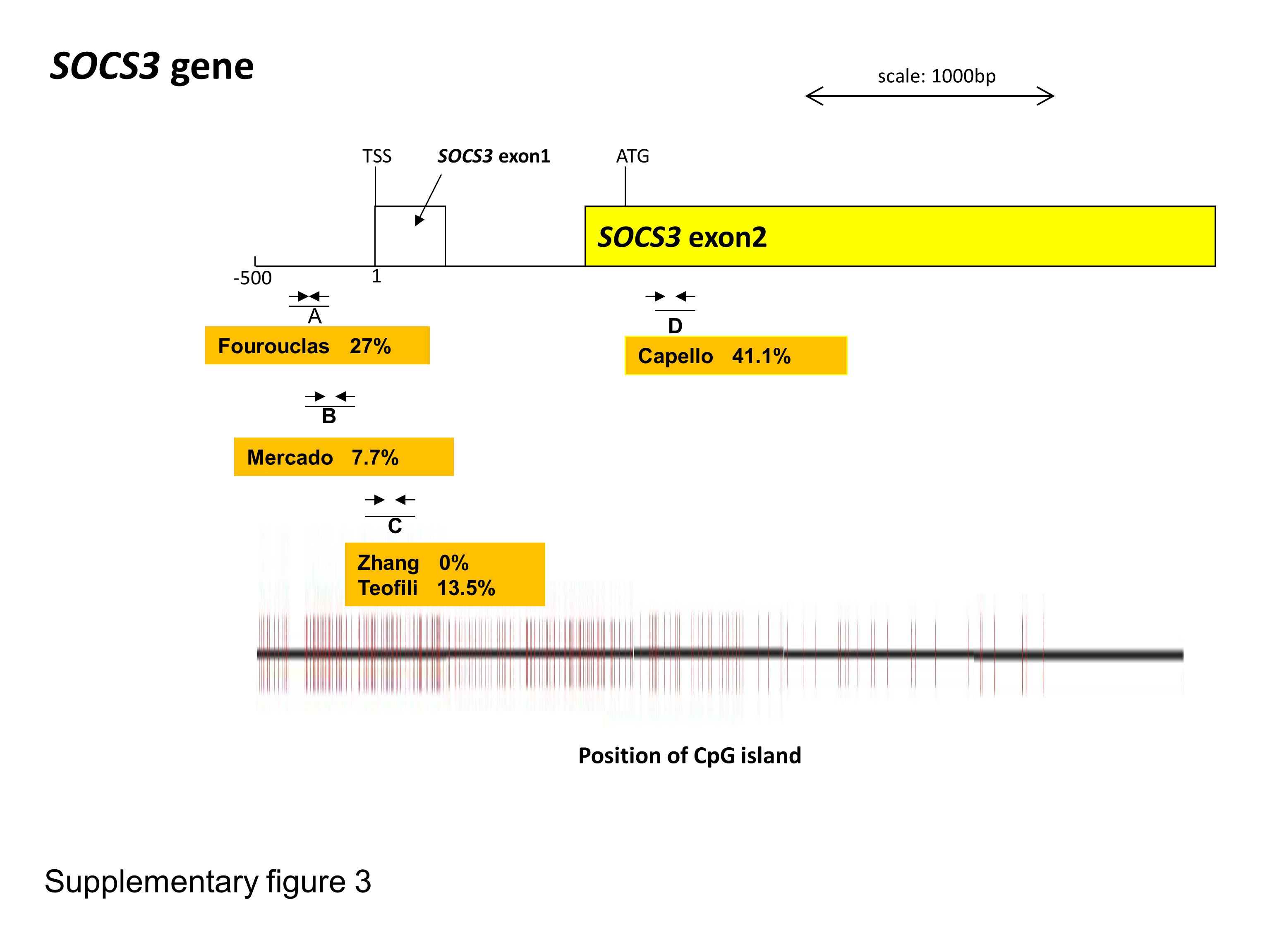

Supplement: Supplementary file 3 [file jcmm0017-1282-SD3.tif]
