# Supplementary material for: Methylation profiling of SOCS1, SOCS2, SOCS3, CISH and SHP1 in Philadelphia-negative myeloproliferative neoplasm
Source: J Cell Mol Med. 2013 Oct 16;17(10):1282–90. doi: 10.1111/jcmm.12103 (PMC4159021; doi:10.1111/jcmm.12103)
Supplement: Supplementary file 4 [file jcmm0017-1282-SD4.doc]

**Legend for Supplementary figure**:

Figure 1. Schematic diagram of the *SOCS1* gene and the positions of primers for MSP. Primers positions are represented by arrows.

Figure 2. Schematic diagram of the *SOCS2* gene and the positions of primers for MSP. Primers positions are represented by arrows.

Figure 3. Schematic diagram of the *SOCS3* gene and the positions of primers for MSP. Primers positions are represented by arrows.

**Supplementary Table : MSP primer sequences and reaction conditions**

| **Gene** | **Forward primer (5’ – 3’)** | **Reverse primer (5’ – 3’)** | **Tm/cycles/Mg^2+^** | **Reference** |
| --- | --- | --- | --- | --- |
| *SOCS1* |  |  |  | Chim *et al,* 2004 |
| M-MSP | GTT GTA GGA TGG GGT CGC GGT CGC | CTA CTA ACC AAA CTA AAA TCC ACA | 58°C/35/2mM |  |
| U-MSP | GTT GTA GGA TGG GGT TGT GGT TGT | CTA CTA ACC AAA CTA AAA TCC ACA | 62°C/40/1.5mM |  |
| *SOCS2*-3’ |  |  |  | NC_000012.11 region  (93963598-93970521) |
| M-MSP | AGT TCG GTT AGA TAG GTA GGG AGT C | GAT CCC TTA AAA ACA AAA AAC GTA | 62°C/36/2mM |  |
| U-MSP | TTT GGT TAG ATA GGT AGG GAG TTG A | AAT CCC TTA AAA ACA AAA AAC ATA | 57°C/33/2mM |  |
| *SOCS2*-5’ |  |  |  | Teofili *et al*, 2008 |
| M-MSP | TTT TAG GAT TTG ATT AAG GGG ATC | TAC GAA AAA TAA ACG TAC AAA AAC G | 55°C /37/2mM |  |
| U-MSP | TTT TTT AGG ATT TGA TTA AGG GGA TT | CAA AAA ATA AAC ATA CAA AAA CAA | 55°C /40/2mM |  |
| *SOCS3* |  |  |  | He *et al*, 2003 |
| M-MSP | TAT ATA TTC GCG AGC GCG GTT T | CGC TAC GCC CAA ATA TTA ACG | 56°C /40/2mM |  |
| U-MSP | TGT GGT GGT TGT TTA TAT ATT TGT GAG TGT GGT T | CAA CCA ACA ATA ACC CAC ACT ACA CCC A | 62°C /35/1.5mM |  |
| *CISH* |  |  |  | NT_006014 region  (1764000-1770000) |
| M-MSP | GTT TAG GAG CGG TCG TTT TAA TAC | TTA CCT TCT AAC ATT CTA CGT CGT T | 58°C /35/2mM |  |
| U-MSP | TTT AGG AGT GGT TGT TTT AAT ATG G | TTA CCT TCT AAC ATT CTA CAT CAT T | 58°C /40/2mM |  |
| *SHP1* |  |  |  | Chim *et al*, 2004 |
| M-MSP | GAA CGT TAT TAT AGT ATA GCG TTC | TCA CGC ATA CGA ACC CAA ACG | 58°C /37/2mM |  |
| U-MSP | GTG AAT GTT ATT ATA GTA TAG TGT TTG G | TTC ACA CAT ACA AAC CCA AAC AAT | 55°C /35/2mM |  |

Tm, annealing temperature; Mg^2+^, concentration of MgCl_2_
